# The mitogenome data of *Holothuria* (*Mertensiothuria*) *leucospilota* (Brandt,1835) from Malaysia

**DOI:** 10.1016/j.dib.2023.108968

**Published:** 2023-02-11

**Authors:** Nur Sabrina Badrulhisham, Siti Najihah Solehin, Ming Gan Han, Puteri Nur Syahzanani Jahari, Faezah Mohd Salleh, Aisyah Mohamed Rehan, Kamarul Rahim Kamarudin

**Affiliations:** a Centre of Research for Sustainable Uses of Natural Resources (SUNR), Faculty of Applied Sciences and Technology (FAST), Universiti Tun Hussein Onn Malaysia (UTHM), Pagoh Campus, Pagoh Education Hub, Muar 84600, Johor Darul Ta'zim, Malaysia; b Department of Biological Sciences, Sunway University, Bandar Sunway, Petaling Jaya 47500, Malaysia; c Department of Biosciences, Faculty of Science, Universiti Teknologi Malaysia, 81310 Johor Bahru, Johor, Malaysia; d Department of Chemical Engineering Technology, Faculty of Engineering Technology (FTK), Universiti Tun Hussein Onn Malaysia (UTHM), Pagoh Campus, 84600 Muar, Johor Darul Ta'zim, Malaysia

**Keywords:** *Holothuria leucospilota*, Mitochondrial genome, Phylogeny, Sea cucumber, Malaysia

## Abstract

White threads fish *Holothuria (Mertensiothuria) leucospilota* (Brandt, 1835) or locally known as *bat puntil* is a neritic marine organism, and it is widely distributed in Indo Pacific. They serve many important roles in ecosystem services and were discovered to contain many bioactive compounds that are useful for medicinal value. However, despite its abundance in Malaysian seawater, there is still a lack of records on *H. leucospilota* mitochondrial genome (mitogenome) from Malaysia. The mitogenome of *H. leucospilota* originating from Sedili Kechil, Kota Tinggi, Johor, Malaysia, is presented here. Whole genome sequencing was successfully sequenced using Illumina NovaSEQ6000 sequencing system and the mitochondrial-derived contigs were assembled using *de novo* approach. The size of the mitogenome is 15,982 bp which consists of 13 protein-coding genes (PCGs), 21 transfer RNAs, and 2 ribosomal RNAs. The overall composition of nucleotide bases was estimated to be 25.8% for T, 25.9% for C, 31.8% for A and 16.5% for G (with *A* + T content of 57.6%). Maximum likelihood phylogenetic tree analysis revealed that the mitochondrial Protein-Coding Genes (PCGs) sequence data from our *H. leucospilota* is closely related to *H. leucospilota* from accession number MK940237 and *H. leucospilota* from accession number MN594790, followed by *H. leucospilota* from accession number MN276190, forming sister group with *H. hilla* (MN163001), known as Tiger tail sea cucumber. The mitogenome of *H. leucospilota* will be valuable for genetic research, mitogenome reference and future conservation management of sea cucumber in Malaysia. The mitogenome data of *H. leucospilota* from Sedili Kechil, Kota Tinggi, Johor, Malaysia is available in the GenBank database repository with accession number ON584426.

**Specifications Table**

**Table:** 

Subject	Omics: Genomics
Specific subject area	Sea cucumber, Mitogenomics
Type of data	Tables: Mitogenome features, Base composition and relative skewness, *H. leucospilota* percentage identity from BLAST nucleotide, NCBI Figures: Mitogenomic circular map, phylogenetic tree analysis, *H. leucospilota* collected specimen FASTA: Mitogenome sequence data
How the data were acquired	The whole genome sequencing was performed using Illumina NovaSEQ6000 (San Diego, CA) sequencing system.
Data format	Raw and analyzed
Description of data collection	Genomic DNA: Favorprep™ Tissue Genomic DNA Extraction Mini Kit (Favorgen, Taiwan); DNA quality check: Nanophotometer® (IMPLEN N50 Touch, Germany) and 2% (w/v) agarose horizontal gel electrophoresis (BIO-RAD); Library preparation: 100 ng DNA was fragmented to 350 bp using a Bioruptor followed by NEB Ultra II library preparation (NEB, Ipswich, MA); Sequencing: Illumina NovaSEQ6000 (San Diego, CA) using a run configuration of 2 × 150 bp; *de novo* assembly: MegaHIT (default setting); mitogenome identification and annotation: MitoZ; Percentage identity: BLAST nucleotide, NCBI; Multiple sequence alignment: Jalview v2.11.2.5; Best-fit evolution model phylogenetic tree: MEGA v11.0 and Jmodeltest v2.1.10; Phylogenetic tree: MEGA v11.0.
Data source location	• Institution: Universiti Tun Hussein Onn Malaysia (UTHM) • City/Town/Region: Johor, Kota Tinggi, Sedili Kechil, Tanjung Sedili beach • Country: Malaysia • Latitude and longitude (and GPS coordinates, if possible) for collected samples/data: Latitude: 1.82611N, Longitude: 104.15869 E
Data accessibility	Repository name: NCBI Sequence Read Archive (SRA) [1] Data identification number: SRS12836453 Direct URL to data: https://www.ncbi.nlm.nih.gov/sra/SRS12836453 Repository name: NCBI Bioproject [2] Data identification number: PRJNA826247 Direct URL to data: https://www.ncbi.nlm.nih.gov/bioproject/PRJNA826247 Repository name: NCBI Biosample [3] Data identification number: SAMN27554787 Direct URL to data: https://www.ncbi.nlm.nih.gov/biosample/SAMN27554787 Repository name: NCBI GenBank [4] Data identification number: ON584426 Direct URL to data: https://www.ncbi.nlm.nih.gov/nuccore/ON584426.1 Repository name: Mendeley Data [5] doi:10.17632/k6nsv8vycc.1. Direct URL to data: https://data.mendeley.com/datasets/k6nsv8vycc

## Value of the Data

• This data will offer the mitogenome sequence of *H. leucospilota* originating from Malaysia, which will be valuable for species identification, molecular taxonomy, species conservation, genetic barcoding and phylogenetics of Malaysian sea cucumber.
• This data can be applied in environmental DNA (eDNA) metabarcoding to analyze ecosystems in non-invasive approaches for biodiversity monitoring.
• This data provides sequences that can be applied for partial gene identification and comparison that benefit researchers to resolve both taxonomic issue and product mislabeling in Malaysian sea cucumber markets.
• This data provides PCGs that are useful in phylogenetic tree construction to improve statistical confidence and better resolution analyses compared to partial gene sequence.
• This data would update and improves genetic documentation of *H. leucospilota* in Malaysia, as well as in public genetic database repository.

## 1 Objective

In Malaysia, *H. leucospilota* (Phylum Echinodermata; Class Holothuroidea; Order Aspidochirotida) is known as *bat puntil, balat hitam, bat hitam* [6] or *patola* [7]. Currently, the species is listed as ‘Least Concern’ on the International Union for Conservation of Nature (IUCN) Red List of Threatened Species status and is considered low value species in markets [8], however, according to a previous report, the species is often vulnerable to overexploitation after high-value sea cucumber species in the fishing zone are depleted [7,8] as there are few to no regulations of the species fished [9]. Therefore, these issues consequently lead the species into a brink of local extinction [7]. Presently, there is still no record of *H. leucospilota* mitogenome from Malaysia. The most recent sourced records of *H. leucospilota* mitogenome obtained from GenBank, NCBI repository are from China [10], [11], [12]. Thus, our objective is to obtain a complete mitogenome of *H. leucospilota* originating from Sedili Kechil, Kota Tinggi, Johor, Malaysia.

## 2 Data Description

**Fig. 1:**
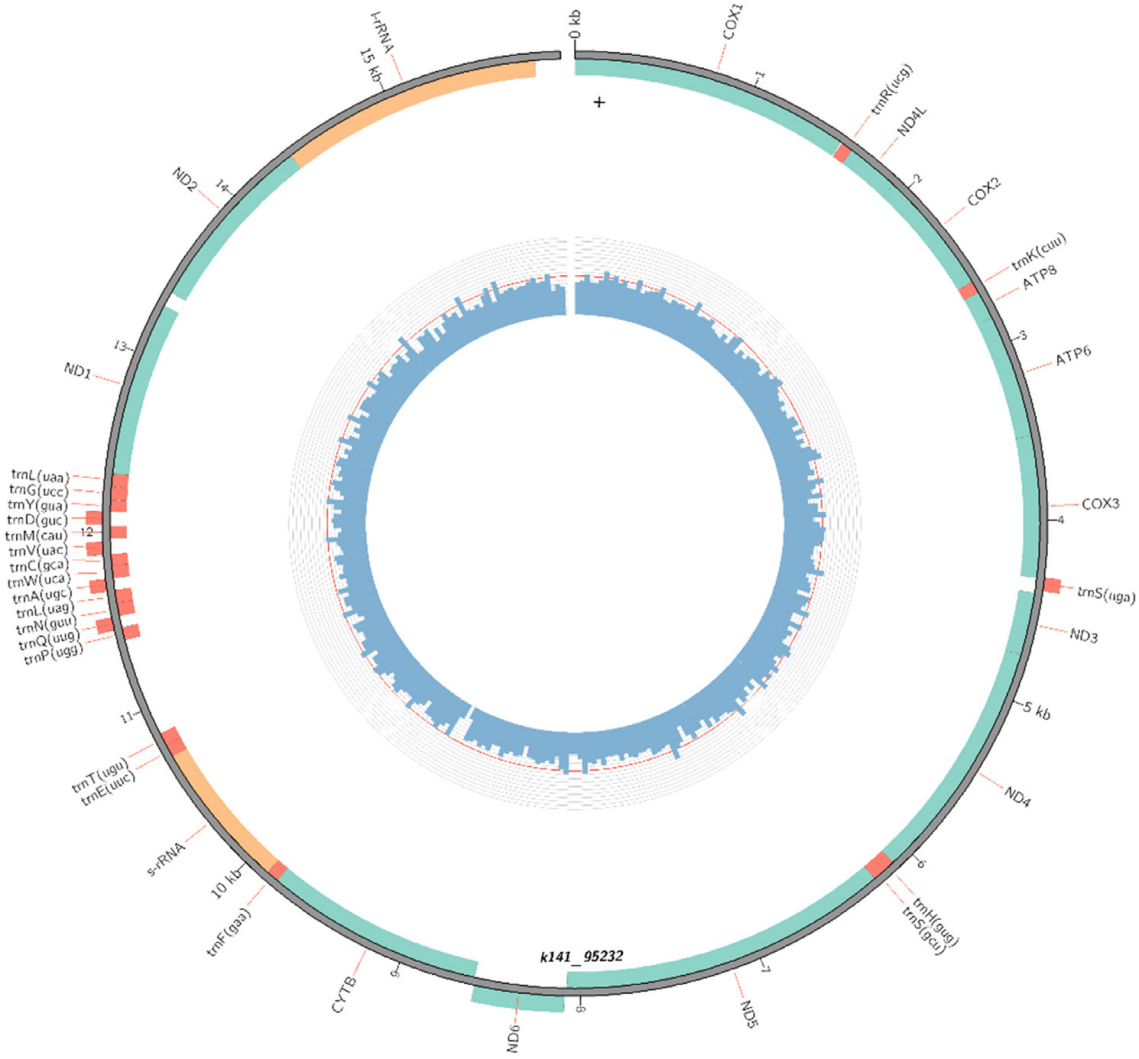
A circular map of *H. leucospilota* 15,982 bp mitogenome specimen collected from Sedili Kechil, Johor, Malaysia generated using MitoZ. The genes encoded at outer side show reverse strand, while the inner side shows forward strand. Green coloured indicates PCGs, orange coloured indicates transfer RNAs, and yellow coloured indicates ribosomal RNAs.

The mitogenome of *H. leucospilota* showed a total length of 15,982 bp which encode 13 protein-coding genes (*COX1, COX2, COX3, ND4L, CYTB, ATP8, ATP6, ND1, ND2, ND3, ND4, ND5, ND6*), 21 transfer RNAs and 2 ribosomal RNAs (*12S rRNA* and *16S rRNA*) (Fig. 1). The overall nucleotide bases composition was estimated to be T 25.8%, C 25.9%, A 31.8% and G 16.5% with A + T content of 57.6%. One gene from transfer RNA was missing (*tRNA-Ile*). The putative control region between *trnT* (*UGU*) and *trnP* (*UGG*) was also not determined possibly due to low coverage during sequencing and difficulty to assemble and sequenced repetitive DNA region [13,14]. Nonetheless, all PCGs are the considered component for phylogenetic reconstruction of sea cucumber species in this study as PCGs illustrate better resolution of functional divergence and speciation [15]. Moreover, whole mitogenomic phylogenetic tree does not indicate a good resolution in the analysis because of the relatively fast evolutionary rate of transfer RNA genes for approximately 7 to 10-fold higher than the genome wide average that would disrupt the construction of the phylogenetic tree [16,17]. Here, the 13 PCGs of *H. leucospilota* are presented with other 36 genes in Table 1 while Table 2 shows the base composition and relative skewness (AT skew and GC skew) of *H. leucospilota* mitogenome. In PCGs, *ND6* gene is the only PCG that encodes at reverse strand while other PCGs encodes at forward strand. Most PCGs have typical mitochondrial start codon *ATG* (Methionine) [18] and the most termination codon is *TAA*, except for *ND4* and *ND6* genes which stop by codon *TAG*.

**Table 1:** Mitogenome features of *H. leucospilota*, where PCGs are represented in bold letters. The direction indicates forward strand (+) and reverse strand (-).

	Position				Start/stop
Gene (codon)	Start	End	Length (bp)	Direction	codon
**COX1**	**1**	**1558**	**1558**	**+**	**ATG/TAA**
trnR (UCG)	1566	1633	68	+	
**ND4L**	**1633**	**1930**	**298**	**+**	**ATG/TAA**
**COX2**	**1930**	**2618**	**689**	**+**	**ATG/AAT**
trnK (CUU)	2618	2686	69	+	
**ATP8**	**2686**	**2851**	**166**	**+**	**ATG/TAA**
**ATP6**	**2844**	**3528**	**685**	**+**	**ATG/TAA**
**COX3**	**3530**	**4313**	**784**	**+**	**ATG/TAA**
trnS (UGA)	4311	4382	72	–	
**ND3**	**4400**	**4745**	**346**	**+**	**ATG/TAA**
**ND4**	**4748**	**6116**	**1369**	**+**	**ATG/TAG**
trnH (GUG)	6106	6173	68	+	
trnS (GCU)	6174	6242	69	+	
**ND5**	**6242**	**8075**	**1834**	**+**	**ATG/TAA**
**ND6**	**8091**	**8580**	**490**	**–**	**ATG/TAG**
**CYTB**	**8588**	**9806**	**1219**	**+**	**ATG/TAA**
trnF (GAA)	9807	9878	72	+	
12S rRNA	9878	10,708	831	+	
trnE (UUC)	10,707	10,776	70	+	
trnT (UGU)	10,777	10,847	71	+	
trnP (UGG)	11,400	11,469	70	+	
trnQ (UUG)	11,465	11,535	71	–	
trnN (GUU)	11,536	11,607	72	+	
trnL (UAG)	11,608	11,680	73	+	
trnA (UGC)	11,679	11,747	69	–	
trnW (UCA)	11,747	11,816	70	+	
trnC (GCA)	11,816	11,877	62	+	
trnV (UAC)	11,876	11,946	71	–	
trnM (CAU)	11,964	12,034	71	+	
trnD (GUC)	12,041	12,111	71	–	
trnY (GUA)	12,111	12,176	66	+	
trnG (UCC)	12,178	12,248	71	+	
trnL (UAA)	12,252	12,323	72	+	
**ND1**	**12,323**	**13,295**	**973**	**+**	**ATG/TAA**
**ND2**	**13,376**	**14,420**	**1045**	**+**	**ATG/TAA**
16S rRNA	14,382	15,841	1460	+

**Table 2:** Base composition and relative skewness (AT skew and GC skew) of *H. leucospilota* mitogenome.

Region	T%	C%	A%	G%	*A* + *T*%	G + C%	AT skew	GC skew
Mitogenome *H. leucospilota*	25.8	25.9	31.8	16.5	57.6	42.4	0.10417	−1.22170
13 PCGs *H. leucospilota*	27.5	26.3	29.9	16.2	57.4	42.5	0.04181	−0.23765
16S gene	20.6	24.3	36.3	18.8	56.9	43.1	0.27592	−0.12761
12S gene	20.2	23.1	35.9	20.7	56.1	43.8	0.27986	−0.05479

The mitogenome PCGs data was compared with other three *H. leucospilota* mitogenome PCGs obtained from GenBank repository, NCBI based on simple pairwise alignment algorithm from BLAST nucleotide (https://blast.ncbi.nlm.nih.gov/) (Table 3). According to the data, the most identical sequence is from [12] (accession number: MK940237), which is 99.56% of identity, followed by [11] (accession number: MN594790) and [10] (accession number: MN276190), which both similarities are 99.40% of identity. Maximum likelihood analysis was implemented in MEGA v11.0 [19] based on 13 concatenated PCGs of 13 individual species of sea cucumber obtained from the GenBank, NCBI repository. General Time Reversible model + Invariant site + Gamma distribution (GTR + I + G) was selected as the best-fit evolution model for the maximum likelihood phylogenetic tree.

**Table 3:** Percentage of identity of *H. leucospilota* (ON584426) simple pairwise alignment sequence obtained from BLAST nucleotide.

PCGs (Percentage of Identity)	Yang, Q (2019) [11] MN594790 (%)	Zhong et al. (2019) [10] MN276190 (%)	Yang et al. (2019) [12] MK940237 (%)
COX1	99.68	99.68	99.49
ND4L	98.99	99.33	98.99
COX2	99.27	99.42	99.56
ATP8	99.39	98.79	98.79
ATP6	99.27	98.54	98.98
COX3	98.60	98.98	98.34
ND3	99.42	98.84	98.84
ND4	99.12	98.98	99.27
ND5	99.40	99.40	99.56
ND6	99.18	99.59	99.18
CYTB	99.86	99.57	99.42
ND1	99.38	99.28	99.38
ND2	99.14	98.95	99.33
Overall PCGs	99.40	99.40	99.56

**Fig. 2:**
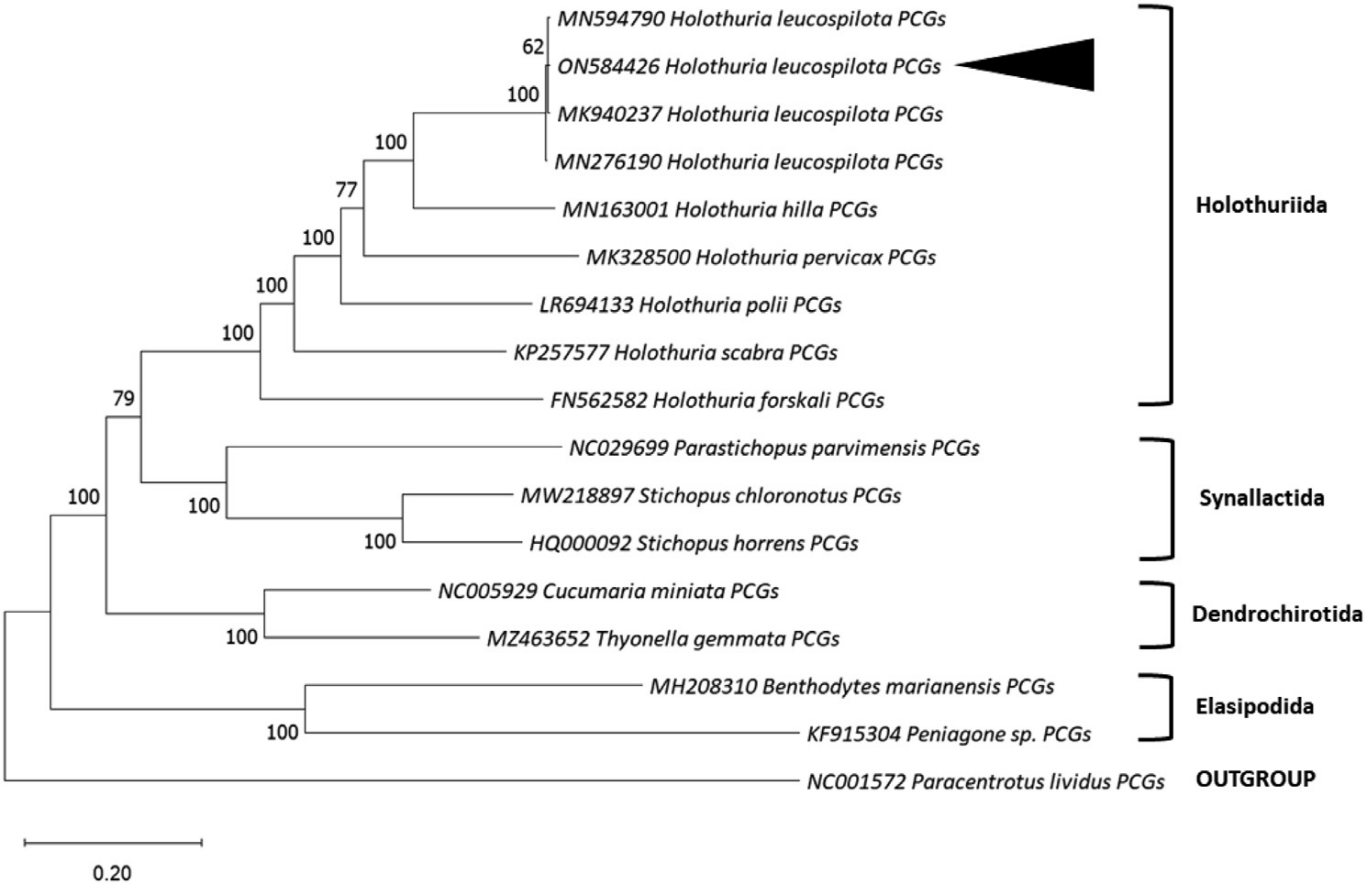
The maximum likelihood phylogenetic tree of 13 concatenated PCGs of sea cucumber with 1000 bootstraps probability. The position of our *H. leucospilota* is marked in solid triangle shape. The number at nodes indicate bootstrap probability. GenBank accession number is listed before the scientific name of the species.

According to the maximum likelihood phylogenetic tree, *H. leucospilota* from Sedili Kechil is clustered together to *H. leucospilota* from accession number MK940237 [12] and *H. leucospilota* from accession number MN594790 [11], followed by *H. leucospilota* from accession number MN276190 [10], which formed an independent branch, in which displayed as a small distinct from other individuals of the same species. *H. leucospilota* is the sister group to *H. hilla* (accession number: MN163001), known as Tiger tail sea cucumber and clustered together with other species from order Holothuriida in a monophyletic clade (Fig. 2).

## 3 Experimental Design, Materials and Methods

### 3.1 Specimen sampling and library preparation

**Fig. 3:**
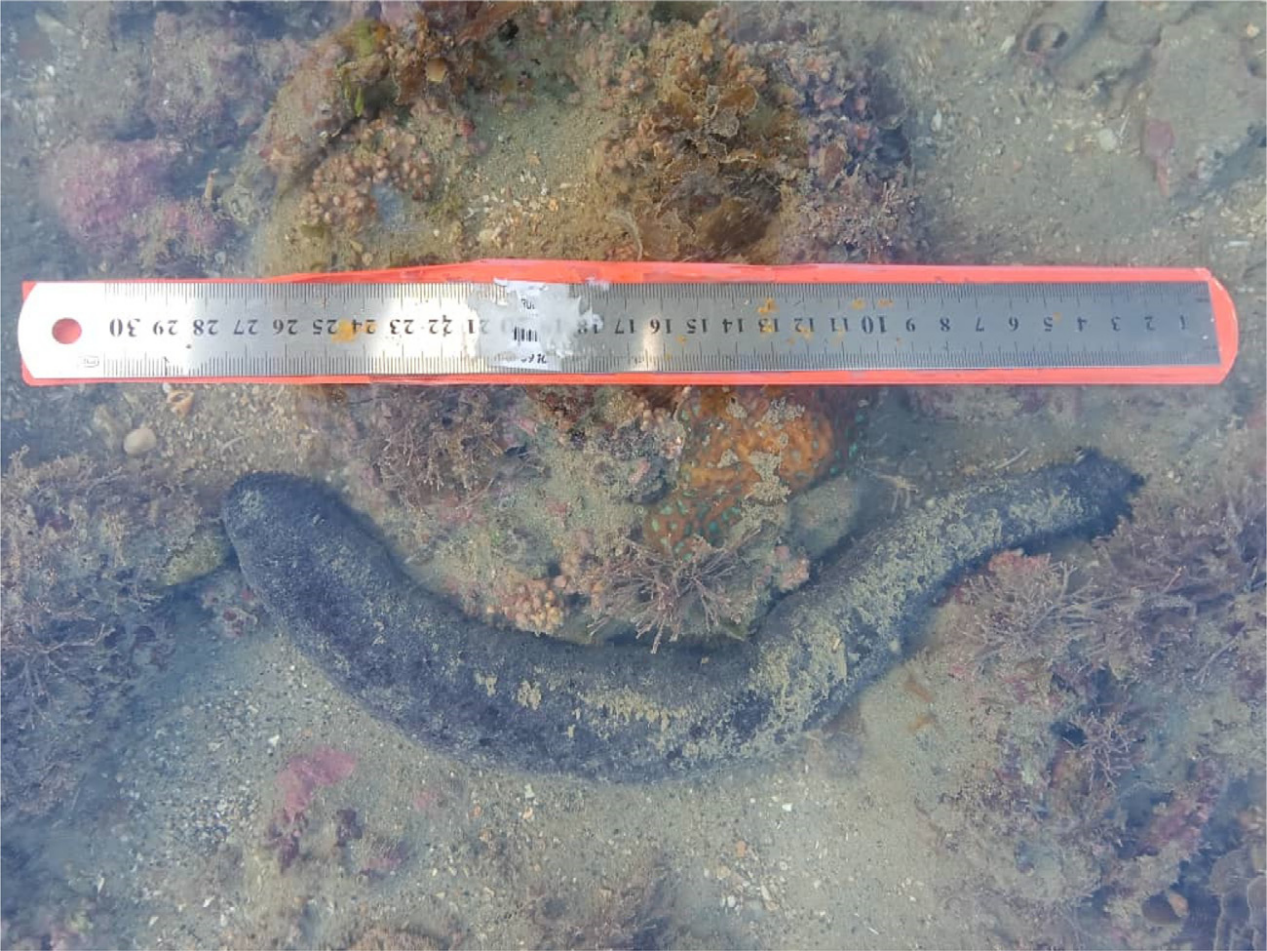
*H. leucospilota* collected from Tanjung Sedili Beach, Sedili Kechil, Kota Tinggi, Johor Darul Ta'zim, Malaysia.

The individual specimen (BioSample number: SAMN27554787 [3]) was collected at intertidal zone of Tanjung Sedili Beach during low tide on November 2021 (Latitude: 1.82611N Longitude: 104.15869E) (Fig. 3). *H. leucospilota* specimen was confirmed its locality by referring previous article [20] and identified based on its feature characteristics and behavior: entirely black-coloured body, cylindrical, elongated snake-like body, moderately tapered at anterior and posterior ends but broader at posterior half, mouth have 20 peltate tentacles [10,9]. The species excreted white sticky threads (Cuvierian tubules) and internal organs from anal openings under stress. The specimen was anesthetized using 5% MgSO_4_ solute with seawater and then preserved in ethyl alcohol (95% ethanol) and stored in 4°C fridge with proper tagging. The total genomic DNA (total gDNA) of *H. leucospilota* specimen was isolated from muscle tissue of the specimen using Favorprep™ Tissue Genomic DNA Extraction Mini Kit (Favorgen, Taiwan) according to manufacturer's instructions with minor modifications. The extracted total gDNA was subjected to Nanophotometer® (IMPLEN N50 Touch, Germany) and 2% (weight/volume) agarose horizontal gel electrophoresis (BIO-RAD) to verify the quantity and quality of total gDNA.

### 3.2 Library preparation and mitogenome assembly

For library preparation, approximately 100 ng of DNA was fragmented to 350 bp using a Bioruptor followed by NEB Ultra II library preparation (NEB, Ipswich, MA) according to the manufacturer's instructions. Whole genome sequencing was performed on an Illumina NovaSEQ6000 (San Diego, CA) using a run configuration of 2 × 150 bp to generate approximately 1 Gb of data for each sample. The generated raw data was deposited in the NCBI Sequence Read Archive (SRA) under accession number: SRS12836453 [1]. Then, the generated raw reads were trimmed with fastp v0.21 [21] for quality check and providing clean data by eliminating low-quality bases and Illumina adapter sequences. The trimmed reads were then assembled into contigs in *de novo* assembler MegaHIT (by default setting) [22]. The mitochondrial-derived contigs were identified, circularised and annotated using MitoZ [23].

### 3.3 Phylogenetic analysis

A maximum likelihood phylogenetic tree was constructed using MEGA v11.0 [19] based on 13 concatenated PCGs of 16 individuals sea cucumber species obtained from GenBank, NCBI repository (https://www.ncbi.nlm. nih.gov/), including our *H. leucospilota* specimen (accession number: ON584426). The multiple sequence was aligned using MAFFT [24] from Jalview v2.11.2.5 [25] and trimmed using MEGA v.11.0 [19]. The phylogenetic tree was constructed using MEGA 11.0 software [19]. General Time reversible + Invariant site + Gamma distribution (GTR + I + G) was selected as best-fit evolutionary model for maximum likelihood phylogenetic tree construction using MEGA v11.0 [19] and Jmodeltest v2.1.10 [26]. *Paracentrotus lividus*, a species of sea urchin (Phylum Echinodermata; Class Echinoidea; Order Camarodonta) was rooted as the outgroup species (accession number: NC001572).

## Ethics Statements

The experiment complied with the ARRIVE guidelines and were carried out in accordance with the U.K. Animals (Scientific Procedures) Act, 1986 and associated guidelines; EU Directive 2010/63/EU for animal experiments; or the National Institutes of Health guide for the care and use of laboratory animals (NIH Publications No. 8023, revised 1978).

## CRediT authorship contribution statement

**Nur Sabrina Badrulhisham:** Conceptualization, Formal analysis, Methodology, Investigation, Data curation, Writing – original draft, Visualization. **Siti Najihah Solehin:** Conceptualization, Software, Methodology, Investigation. **Ming Gan Han:** Conceptualization, Resources, Formal analysis, Software, Data curation, Methodology, Writing – review & editing. **Puteri Nur Syahzanani Jahari:** Conceptualization, Software, Methodology, Writing – review & editing. **Faezah Mohd Salleh:** Conceptualization, Software, Methodology, Writing – review & editing. **Aisyah Mohamed Rehan:** Funding acquisition, Methodology. **Kamarul Rahim Kamarudin:** Funding acquisition, Conceptualization, Software, Investigation, Resources, Methodology, Writing – review & editing, Supervision.

## Declaration of Competing Interest

The authors declare that they have no known competing financial interests or personal relationships that could have appeared to influence the work reported in this paper.

## Data Availability

The Mitogenome Data of Holothuria (Mertensiothuria) leucospilota (Brandt, 1835) from Malaysia. (Original data) (Mendeley Data).
